# Laminarin Pretreatment Provides Neuroprotection against Forebrain Ischemia/Reperfusion Injury by Reducing Oxidative Stress and Neuroinflammation in Aged Gerbils

**DOI:** 10.3390/md18040213

**Published:** 2020-04-15

**Authors:** Joon Ha Park, Ji Hyeon Ahn, Tae-Kyeong Lee, Cheol Woo Park, Bora Kim, Jae-Chul Lee, Dae Won Kim, Myoung Cheol Shin, Jun Hwi Cho, Choong-Hyun Lee, Soo Young Choi, Moo-Ho Won

**Affiliations:** 1 Department of Anatomy, College of Korean Medicine, Dongguk University, Gyeongju, Gyeongbuk 38066, Korea; jh-park@dongguk.ac.kr; 2 Department of Biomedical Science and Research Institute for Bioscience and Biotechnology, Hallym University, Chuncheon, Gangwon 24252, Korea; jh-ahn@hallym.ac.kr (J.H.A.); xorud312@naver.com (T.-K.L.); 3 Department of Neurobiology, School of Medicine, Kangwon National University, Chuncheon, Gangwon 24341, Korea; flfhflfh@naver.com (C.W.P.); nbrkim17@gmail.com (B.K.); anajclee@kangwon.ac.kr (J.-C.L.); 4 Department of Biochemistry and Molecular Biology, and Research Institute of Oral Sciences, College of Dentistry, Gangnung-Wonju National University, Gangneung, Gangwon 25457, Korea; kimdw@gwnu.ac.kr; 5 Department of Emergency Medicine, and Institute of Medical Sciences, Kangwon National University Hospital, School of Medicine, Kangwon National University, Chuncheon, Gangwon 24341, Korea; dr10126@naver.com (M.C.S.); cjhemd@kangwon.ac.kr (J.H.C.); 6 Department of Pharmacy, College of Pharmacy, Dancook University, Cheonan, Chungcheongnam 31116, Korea; anaphy@dankook.ac.kr

**Keywords:** laminarin, aging, transient cerebral ischemia, neuroprotection, oxidative stress, neuroinflammation

## Abstract

Laminarin is a polysaccharide isolated from brown algae that has various biological and pharmacological activities, such as antioxidant and anti-inflammatory properties. We recently reported that pretreated laminarin exerted neuroprotection against transient forebrain ischemia/reperfusion (IR) injury when we pretreated with 50 mg/kg of laminarin once a day for seven days in adult gerbils. However, there have been no studies regarding a neuroprotective effect of pretreated laminarin against IR injury in aged animals and its related mechanisms. Therefore, in this study, we intraperitoneally inject laminarin (50 mg/kg) once a day to aged gerbils for seven days before IR (5-min transient ischemia) surgery and examine the neuroprotective effect of laminarin treatment and the mechanisms in the gerbil hippocampus. IR injury in vehicle-treated gerbils causes loss (death) of pyramidal neurons in the hippocampal CA1 field at five days post-IR. Pretreatment with laminarin effectively protects the CA1 pyramidal neurons from IR injury. Regarding the laminarin-treated gerbils, production of superoxide anions, 4-hydroxy-2-nonenal expression and pro-inflammatory cytokines [interleukin(IL)-1β and tumor necrosis factor-α] expressions are significantly decreased in the CA1 pyramidal neurons after IR. Additionally, laminarin treatment significantly increases expressions of superoxide dismutase and anti-inflammatory cytokines (IL-4 and IL-13) in the CA1 pyramidal neurons before and after IR. Taken together, these findings indicate that laminarin can protect neurons from ischemic brain injury in an aged population by attenuating IR-induced oxidative stress and neuroinflammation.

## 1. Introduction

Transient ischemia in the brain occurs when the blood supply to part of the brain or the whole brain is briefly interrupted by occlusion of regional arteries or by cardiac ischemia [1,2]. Transient brain ischemia results in irreversible and persistent damage to the brain parenchyma [3,4]. Globally, transient ischemia-induced brain injury is a major cause of death or long-term disability [5], showing that the brain injury is triggered by complex mechanisms [6,7]. Among the complex mechanisms, oxidative stress and neuroinflammation have been regarded as major contributors to the pathogenesis of ischemic brain injury following transient brain ischemia [8,9,10]. Therefore, inhibition of oxidative stress and neuroinflammation has been a focus of studies on developing neuroprotection against transient ischemia-induced brain injury [11,12] and neurodegenerative disease [13].

Many studies have been conducted to develop therapeutic candidates against ischemic brain injury using experimental animals; however, these studies have been carried out in adult animals [14,15,16]. Brain ischemia (ischemic stroke) is one of the most important age-related diseases. Approximately one third of patients with ischemic stroke are in the aged population [17]. Due to a longer life expectancy, the incidence of brain ischemia will increase further over time, and aging has been known as one of the major risk factors affecting ischemic brain injury [18,19,20]. Aged brains are more susceptible to ischemic brain injury, and protective efficacy against ischemic injury is low in the aged population [21]. Thus, we need to assess therapeutic strategies against ischemic brain injury in aged experimental animals.

Medicinal plant-derived natural compounds have received considerable attention as potential sources of therapeutic candidates for the prevention and treatment of neurological diseases, including brain ischemia, because they display a wide spectrum of biological properties [22,23]. Marine algae-derived compounds also have been shown to protect against brain ischemic injury. Fucoidan, for example, a type of polysaccharide extracted from various species of brown algae, shows strong neuroprotective effects against ischemic brain injury in animal models of brain ischemia [24,25,26,27]. Additionally, we recently reported that laminarin, a water-soluble polysaccharide derived from the brown algae *Laminaria digitate*, showed neuroprotection against IR brain injury in adult gerbils [28]. Laminarin exhibits biofunctional properties, such as anti-apoptotic [29], anti-oxidant [30], anti-inflammatory [31], and anti-cancer [32] activities. 

However, to the best of our knowledge, there have been few studies about the neuroprotective effects of laminarin against ischemic brain injury in aged experimental animals, although there is a high incidence of brain ischemia in the aged population. Additionally, the underlying mechanisms of the neuroprotective effects of laminarin against ischemic brain injury have not been fully addressed. Thus, our objective in this study is to investigate the neuroprotective effect and mechanisms of laminarin as a potential neuroprotective agent in the hippocampus of aged gerbils following brain IR injury. The hippocampus is well known as one of the brain regions most vulnerable to transient brain ischemia [33,34]. Specifically, extensive loss of pyramidal neurons takes place in the hippocampal cornu ammonis 1 (CA1) region over several days after transient ischemia in both humans and experimental animals [3,7,35].

## 2. Results

### 2.1. Protection of Neurons from IR Injury by Laminarin

#### 2.1.1. Neuronal Nuclear Antigen (NeuN) Immunoreactive (^+^) Cells

**Figure 1:**
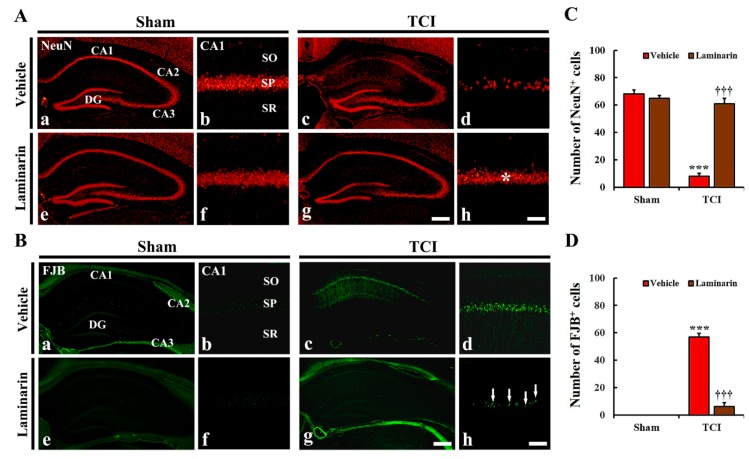
(**A**,**B**) Representative images of NeuN immunofluorescence (**A**) and FJB histofluorescence staining (**B**) in CA1 of the vehicle-sham (**a**,**b**), vehicle-IR (**c**,**d**), laminarin-sham (**e**,**f**), and laminarin-IR (**g**,**h**) groups at five days after IR. Regarding the vehicle-IR group, a few NeuN^+^ and many FJB^+^ cells are shown in the stratum pyramidal (SP). However, in the laminarin-IR group, numerous NeuN^+^ cells (asterisk) and a few FJB^+^ cells (arrows) are shown in the SP. CA, cornu ammonis; DG, dentate gyrus; FJB, Fluoro-Jade B; NeuN, nuclear antigen; SO, stratum oriens; SR, stratum radiatum. Scale bar = 400 μm (**a**,**c**,**e**,**g**), 50 μm (**b**,**d**,**f**,**h**). (**C**,**D**) Mean number of NeuN^+^ (**C**) and FJB^+^ cells (**D**) in CA1. The bars indicate means ± SEM (*n* = 7/group; *** *p* < 0.001 versus each sham group, ^†††^ *p* < 0.001 versus vehicle-IR group).

During the vehicle-sham and laminarin-sham groups, NeuN immunofluorescence was easily detected in intact neurons in all hippocampal subregions (Figure 1A(a,e)), and this finding was not different from that in the control group (data not shown). Regarding the hippocampus proper [cornu ammonis 1(CA1)–CA3], pyramidal cells, which consist of the striatum pyramidal, showed strong NeuN immunofluorescence (Figure 1A(a,b,e,f)). 

Concerning the vehicle-IR group, NeuN^+^ pyramidal cells were significantly decreased in number in CA1, but not in CA2/3, at five days post-IR (Figure 1A(c,d)). Then, the number of NeuN^+^ CA1 pyramidal cells was 8.4 ± 2.2 cells/200 × 200 μm (Figure 1C). However, in the laminarin-IR group, a considerable number of NeuN^+^ CA1 pyramidal cells (61.3 ± 4.1 cells/200 × 200 μm) was observed, compared to that in the vehicle-IR group (Figure 1A(g,h),C). This finding means that pretreated laminarin protected hippocampal CA1 pyramidal neurons from 5-min IR in aged gerbils.

#### 2.1.2. Fluoro-Jade B (FJB)^+^ Cells

Concerning the vehicle-sham and laminarin-sham groups, FJB^+^ cells, which are dead cells, were not detected in the hippocampus (Figure 1B(a,b,e,f)). Also, similar results were obtained in the control group of animals (data not shown).

Regarding the vehicle-IR group, numerous FJB^+^ cells were shown in the stratum pyramidal of CA1 at five days post-IR (Figure 1B(c,d)), showing that the number of FJB^+^ CA1 pyramidal cells was 57.6 ± 2.5 cells/200 × 200 μm (Figure 1D). Concerning the laminarin-IR group, only a few FJB^+^ CA1 pyramidal cells (6.4 ± 3.3 cells/200 × 200 μm) were shown in CA1, compared to those in the vehicle-IR group (Figure 1B(g,h),D). 

### 2.2. Increases of Superoxide Dismutase (SODs) Expression by Laminarin

**Figure 2:**
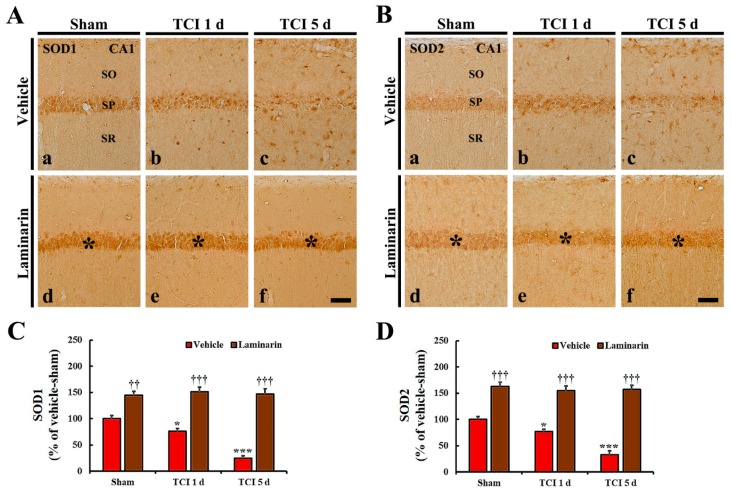
(**A**,**B**) Images of immunochemistry for SOD1 (**A**) and SOD2 (**B**) in CA1 of the vehicle-sham (**a**), vehicle-IR (**b**,**c**), laminarin-sham (**d**), and laminarin-IR (**e**,**f**) groups at one day (**b**,**e**) and five days (**c**,**f**) after IR. Regarding the vehicle-IR group, SOD1 and SOD2 immunoreactivity are significantly increased with time in the stratum pyramidale (SP). Concerning the laminarin-sham group, SOD1 and SOD2 immunoreactivity in the SP (asterisks) are significantly higher than that in the vehicle-sham group. Regarding the laminarin-IR group, both immunoreactivities (asterisks) are sustained until five days post-IR. SOD, superoxide dismutase; SO, stratum oriens; SR, stratum radiatum. Scale bar = 50 μm. (**C**,**D**) Relative immunoreactivity (RI) of SOD1 (**C**) and SOD2 (**D**) immunoreactivity in CA1 pyramidal cells. The bars indicate the means ± SEM (*n* = 7/group; * *p* < 0.05, *** *p* < 0.001 versus each sham group, ^††^ *p* < 0.01, ^†††^ *p* < 0.001 versus vehicle-IR group).

Regarding the vehicle-sham group, copper-zinc SOD (SOD1) and manganese SOD (SOD2) immunoreactivity were easily shown in CA1 pyramidal cells (Figure 2A(a),B(a)). Concerning the vehicle-IR group, SOD1 and SOD2 immunoreactivity in the CA1 pyramidal cells were significantly decreased at one day post-IR (by about 24% and 22%, respectively) compared to that in the vehicle-sham group (Figure 2A(b),B(b),C,D), and, at five days post-IR, SOD1 and SOD2 immunoreactivity were significantly decreased further (about 25% and 34% of the vehicle-sham group, respectively) (Figure 2A(c),B(c),C,D).

Seen in the laminarin-sham group, SOD1 and SOD2 immunoreactivity in CA1 pyramidal cells were significantly higher (about 145% and 163%, respectively) than that in the vehicle-sham group (Figure 2A(d),B(d),C,D). Interestingly, in the laminarin-IR group, the increased SOD1 and SOD2 immunoreactivity were sustained until five days post-IR (Figure 2A(e,f),B(e,f),C,D).

### 2.3. Attenuation of IR-Induced Oxidative Stress by Laminarin

#### 2.3.1. Dihydroethidium (DHE) Fluorescence

**Figure 3:**
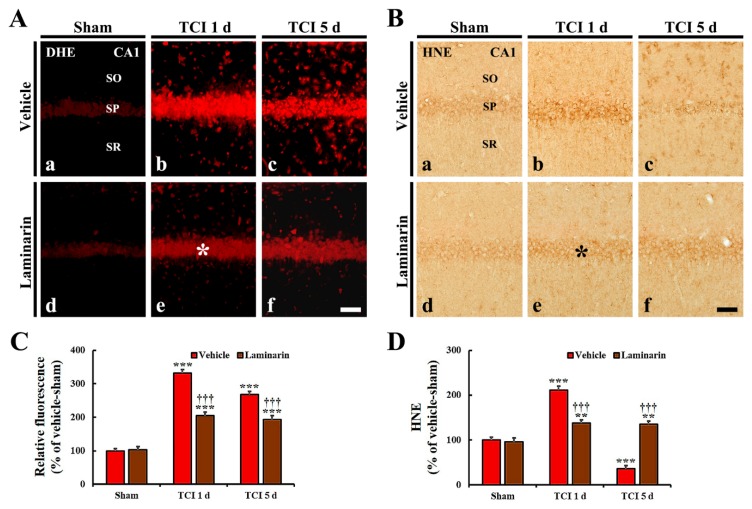
(**A**,**B**) DHE fluorescence staining (**A**) and HNE immunohistochemistry (**B**) in CA1 of the vehicle-sham (**a**), vehicle-IR (**b**,**c**), laminarin-sham (**d**), and laminarin-IR (**e**,**f**) groups at one day (**b**,**e**) and five days (**c**,**f**) after IR. Regarding the vehicle-IR group, DHE fluorescence and HNE immunoreactivity in the stratum pyramidale (SP) are significantly increased at one day post-IR and decreased at five days post-IR. Concerning the laminarin-IR group, DHE fluorescence (asterisk) and HNE immunoreactivity (asterisk) at one day post-IR are significantly lower than those in the vehicle-IR group, and, at five days post-IR, DHE fluorescence and HNE immunoreactivity are maintained. Dihydroethidium, DHE; 4-hydroxy-2-nonenal, HNE; SO, stratum oriens; SR, stratum radiatum. Scale bar = 50 μm. (**C**,**D**) Relative immunoreactivity (RI) of DHE fluorescence (**C**) and HNE immunoreactivity (**D**) in CA1 pyramidal cells. The bars indicate the mean ± SEM (*n* = 7/group; ** *p* < 0.01, *** *p* < 0.001 versus each sham group, ^†††^ *p* < 0.001 versus vehicle-IR group).

Weak DHE fluorescence was detected in CA1 pyramidal cells of the vehicle-sham group (Figure 3Aa). Regarding the vehicle-IR group, DHE fluorescence intensity in the CA1 pyramidal cells was significantly increased by about 333% at one day post-IR and by about 269% at five days post-IR, compared with that in the vehicle-sham group (Figure 3A(b,c),C). Particularly, at one and five days post-IR, strong DHE fluorescence was shown in many non-pyramidal cells located in strata oriens and radiatum (Figure 3A(b,c)).

Concerning the laminarin-sham group, DHE fluorescence and its intensity in CA1 pyramidal cells were not different from those in the vehicle-sham group (Figure 3A(d),C). However, in the laminarin-IR group, DHE fluorescence intensity at one day post-IR was significantly lower compared with that in the vehicle-IR group (about 62% of the vehicle-IR group), and the DHE fluorescence intensity was sustained until five days post-IR (Figure 3A(e,f),C).

#### 2.3.2. 4-Hydroxy-2-Nonenal (HNE) Immunoreactivity

Regarding the vehicle-sham group, weak HNE immunoactivity was detected in CA1 pyramidal cells (Figure 3B(a)). Concerning the vehicle-IR group, HNE immunoreactivity in the CA1 pyramidal cells was significantly increased (by about 212%) at one day post-IR compared with that of the vehicle-sham group and, at five days post-IR, its immunoreactivity was very low because the CA1 pyramidal cells were dead by IR (Figure 3B(b,c),D).

Regarding the laminarin-sham group, HNE immunoreactivity in CA1 pyramidal cells was similar to that in the vehicle-sham group (Figure 3B(d),D). Concerning the laminarin-IR group, HNE immunoreactivity in the CA1 pyramidal cells was significantly low at one day post-IR compared with that in the vehicle-IR group (about 73% of the vehicle-IR group), and its immunoreactivity was not altered until five days post-IR (Figure 3B(e,f),D). 

### 2.4. Reduction of IR-Induced Neuroinflammation by Laminarin

#### 2.4.1. Pro-Inflammatory Cytokine Immunoreactivities

**Figure 4:**
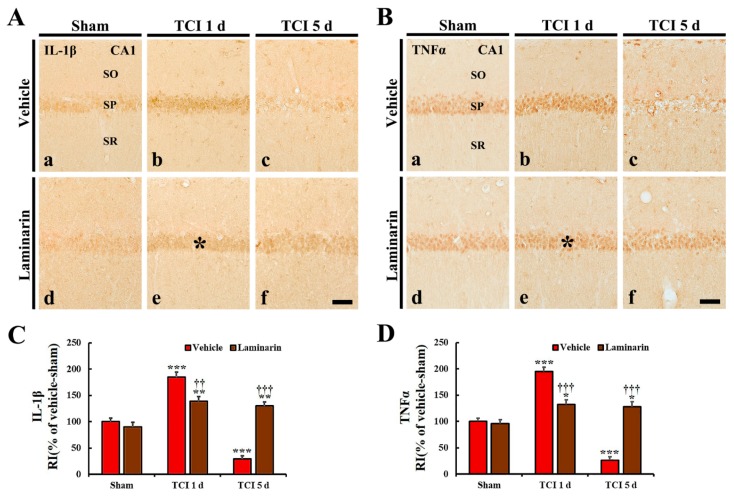
(**A**,**B**) Immunochemistry for IL-1β (**A**) and TNFα (**B**) in CA1 of the vehicle-sham (**a**), vehicle-IR (**b**,**c**), laminarin-sham (**d**), and laminarin-IR (**e**,**f**) groups at one day (**b**,**e**) and five days (**c**,**f**) after IR. Regarding the vehicle-IR group, IL-1β and TNFα immunoreactivities are significantly increased in the stratum pyramidale (SP) (asterisks) and hardly shown at five days post-IR. Concerning the laminarin-IR group, IL-1β and TNFα immunoreactivities in the SP (asterisks) are significantly lower than those in the vehicle-IR group at one day post-IR and not changed at five days post-IR. Interleukin, IL; tumor necrosis factor, TNF; SO, stratum oriens; SR, stratum radiatum. Scale bar = 50 μm. (**C**,**D**) Relative immunoreactivity (RI) of IL-1β (**C**) and TNFα (**D**) immunoreactivity in CA1 pyramidal cells. The bars indicate the means ± SEM (*n* = 7/group; * *p* < 0.05, ** *p* < 0.01, *** *p* < 0.001 versus each sham group, ^††^ *p* < 0.01, ^†††^ *p* < 0.001 versus vehicle-IR group).

Interleukin (IL)-1β and tumor necrosis factor (TNF)α immunoreactivities in the vehicle-sham group were observed in CA1 pyramidal cells (Figure 4A(a),B(a)). Regarding the vehicle-IR group, IL-1β and TNFα immunoreactivities in the CA1 pyramidal cells were significantly increased (by about 186% and 195%, respectively) at one day post-IR compared with that in the vehicle-sham group (Figure 4A(b),B(b),C,D). Occurring by five days post-IR, each immunoreactivity was very low in CA1 pyramidal cells due to damage to the CA1 pyramidal cells by IR (Figure 4A(c),B(c),C,D). 

Regarding the laminarin-sham group, IL-1β and TNFα immunoreactivities in CA1 pyramidal cells were similar to those in the vehicle-sham group (Figure 4A(d),B(d),C,D). However, in the laminarin-IR group, IL-1β and TNFα immunoreactivities in the CA1 pyramidal cells were significantly lower (about 43% and 73%, respectively) at one day post-IR than those in the vehicle-IR group (Figure 4A(e),B(e),C,D), and each immunoreactivity was not changed until five days post-IR (Figure 4A(f),B(f),C,D). 

#### 2.4.2. Anti-Inflammatory Cytokine Immunoreactivities 

**Figure 5:**
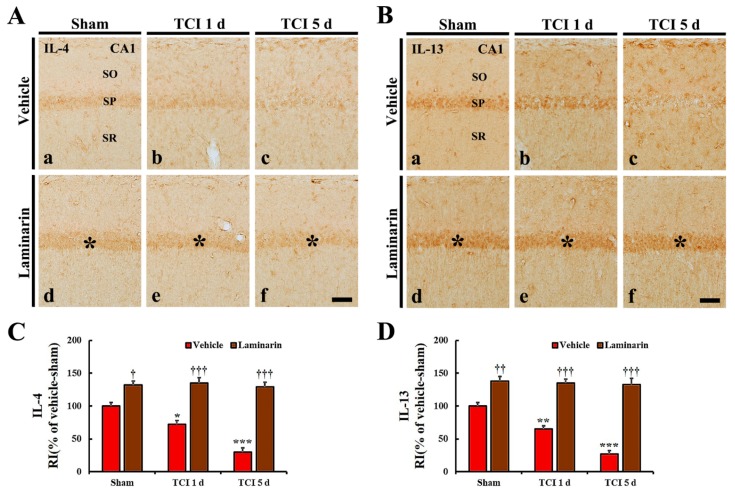
(**A**,**B**) Immunochemistry for IL-4 (**A**) and IL-13 (**B**) in CA1 of the vehicle-sham (**a**), vehicle-IR (**b**,**c**), laminarin-sham (**d**), and laminarin-IR (**e**,**f**) groups at one day (**b**,**e**) and five days (**c**,**f**) after IR. Regarding the vehicle-IR group, IL-4 and IL-13 immunoreactivities in the stratum pyramidale (SP) are gradually decreased after IR. Concerning the laminarin-sham group, IL-4 and IL-13 immunoreactivities in the SP (asterisks) are high compared with those in the vehicle-sham group, and the increased immunoreactivities in the SP (asterisks) are maintained until five days post-IR. IL, interleukin; SO, stratum oriens; SR, stratum radiatum. Scale bar = 50 μm. (**C**,**D**) Relative immunoreactivity (RI) of IL-4 (**C**) and IL-13 (**D**) immunoreactivity in CA1 pyramidal cells. The bars indicate the means ± SEM (*n* = 7/group; * *p* < 0.05, *** *p* < 0.001 versus each sham group, ^†^ *p* < 0.05, ^††^ *p* < 0.01, ^†††^ *p* < 0.001 versus vehicle-IR group).

IL-4 and IL-13 immunoreactivities were found in CA1 pyramidal cells in the vehicle-sham group (Figure 5A(a),B(a)). Regarding the vehicle-IR group, both IL-4 and IL-13 immunoreactivities in the CA1 pyramidal cells were significantly reduced (about 72% and 64%, respectively) at one day post-IR compared with that in the vehicle-sham group (Figure 5A(b),B(b),C,D) and, at five days post-IR, both immunoreactivities were very low due to damage (death) to the CA1 pyramidal cells by IR (Figure 5A(c),B(c),C,D). 

Concerning the laminarin-sham group, IL-4, and IL-13 immunoreactivities in CA1 pyramidal cells were significantly higher (about 132% and 138%, respectively) than that in the vehicle-sham group (Figure 5A(d),B(d),C,D). Regarding the laminarin-IR group, the increased IL-4 and IL-13 immunoreactivities in the CA1 pyramidal cells were maintained at one and five days post-IR (Figure 5A(e,f),B(e,f),C,D).

## 3. Discussion

Pretreatment with polysaccharides isolated from brown algae elicits neuroprotective effects against ischemic brain injuries. Pretreated fucoidan, for example, significantly reduces cerebral infarction size following transient focal cerebral ischemia induced by middle cerebral artery occlusion (MCAO)in adult rats [25] and protects pyramidal neurons in the hippocampal CA1 from IR injury in an adult gerbil model of transient global forebrain ischemia [27]. Furthermore, we recently reported that laminarin pretreatment had a strong neuroprotection in the hippocampal CA1 against IR injury in an adult gerbil model of transient global forebrain ischemia [28]. However, the above-mentioned studies were done in adult animal models; these studies have a limitation in not addressing ischemic insults in the aged population with a higher risk of ischemic stroke. Thus, in this study, we evaluated how pretreatment with laminarin protects neurons in an aged gerbil hippocampus following IR by using NeuN immunofluorescence and FJB histofluorescence staining. We found that pretreatment with laminarin effectively protected CA1 pyramidal neurons from IR injury. This result indicates that laminarin could be applied as a possible candidate for prevention of ischemic stroke in the aged population.

It has been reported that the aged brain is sensitive to oxidative stress and neuroinflammation [36,37]. A growing number of studies also have suggested that oxidative stress caused by excessive production of reactive oxygen species (ROS) and neuroinflammation mediated by activation of glial cells and release of inflammatory mediators are major factors leading to neuronal death following cerebral ischemia [8,38]. Based on the above-mentioned studies, researchers have demonstrated that treatments with pharmacological agents, including natural products, alleviate ischemia-induced oxidative stress and neuroinflammation, and contribute strongly to neuroprotection against ischemic brain injury [27,39,40]. During this study, we used pretreatment of gerbils with laminarin and found that SOD1 and SOD2, as endogenous antioxidant enzymes, were markedly increased in CA1 pyramidal neurons before and after IR induction. It has been suggested that increased endogenous antioxidant enzymes prevent the accumulation of ROS [41] and can protect against ischemic injuries [42]. To investigate whether increased SODs protect neurons from IR injury, we examined levels in DHE (a probe to detect superoxide anions) and HNE (a product of lipid peroxidation), as indicators of oxidative stress in the laminarin-IR group and found they were significantly more alleviated in the CA1 pyramidal neurons than those in the vehicle-IR group. This finding means that IR-induced oxidative stress is significantly reduced by laminarin preconditioning.

Here, we found that levels of pro-inflammatory cytokines (IL-1β and TNFα) were significantly increased in the CA1 pyramidal neurons in the vehicle-IR group after IR but, in the laminarin-IR group, the increased levels of IL-1β and TNFα were significantly reduced after IR. Furthermore, anti-inflammatory cytokines (IL-4 and IL-13), which are involved in the resolution of neuroinflammation [43], were significantly increased in the CA1 pyramidal neurons in the laminarin-IR group before and after IR. 

Although the protective effects of laminarin against IR-induced oxidative stress and neuroinflammation have not been addressed yet in aged brains following ischemic insults, Yao et al. (2018) recently reported that laminarin effectively delayed the aging of oocytes and improved the quality of aged oocytes by reducing the ROS level and increasing the glutathione level. Additionally, Cheng et al. (2011) reported that laminarin significantly attenuated sepsis-induced oxidative damage in the lung of the rat by reducing the malondialdehyde (MDA, an end product of lipid peroxidation) level and increasing the activities of endogenous antioxidant enzymes, such as SODs, catalase, and glutathione peroxidase. Regarding the case of inflammation, Neyrinck et al. (2007) showed that laminarin protected against LPS-induced hepatotoxicity in rats via a decrease in the serum monocyte number, nitrite, and TNFα. Additionally, we recently reported that laminarin pretreatment strongly attenuated activations of astrocytes and microglia in the hippocampal CA1 after brain IR in gerbils [28]. Thus, together with our and other previous studies, our current findings potentially suggest that alleviation of oxidative stress and neuroinflammation in the ischemic hippocampal CA1 of aged gerbils by pretreatment with laminarin contributes to neuroprotection against IR injury.

Here, we investigated IR-induced changes of oxidative stress, antioxidants (SOD1 and SOD2), and inflammatory cytokines (IL-1β, TNFα, IL-4, and IL-13) in the hippocampal CA1 region by immunohistochemistry. Regarding quantitative analyses, these factors will be examined by western blot or real-time polymerase chain reaction in the future study.

To summarize, our findings provide clear evidences that pretreatment with laminarin protects CA1 pyramidal neurons in the hippocampus from IR injury in an aged gerbil model of transient global forebrain ischemia. This effect might be closely related to attenuation of oxidative stress and neuroinflammation in the ischemic CA1 following laminarin pretreatment. Therefore, laminarin can be used as a potential candidate for attenuating ischemic brain injury in the aged population in the future, although further studies are needed to investigate much more specific molecular mechanisms.

## 4. Materials and Methods

### 4.1. Experimental Groups and Laminarin Pretreatment

Aged male gerbils (age 22–24 months [44]; body weight 80–90 g) were supplied by the Experimental Animal Center, Kangwon National University (Chuncheon, Gangwon, Korea). Animal care and handling followed the guidelines of current international laws and policies, which are in the NIH Guide for the Care and Use of Laboratory Animals (The National Academies Press, 8th Ed., 2011), and the protocol of this experiment was approved (approval number, KW- KW-200113-1) by the Institutional Animal Care and Use Committee (IACUC) of Kangwon National University.

A total of 53 gerbils were randomly assigned to five groups: (1) control group (*n* = 5); (2) vehicle-sham group (*n* = 10), which was treated with vehicle (saline) and not subjected to IR; (3) vehicle-IR group (*n* = 14), which was treated with vehicle and subjected to IR; (4) laminarin-sham group (*n* = 10), which was treated with 50 mg/kg of laminarin and not subjected to IR; (5) laminarin-IR group (*n* = 14), which was treated with 50 mg/kg of laminarin and subjected to IR. The gerbils in the experimental groups were sacrificed at 1 day and 5 days after IR surgery. The dosage and duration of laminarin (Sigma–Aldrich, Poole, Dorset, UK) treatment were selected based on the results of our previous study [28]. The vehicle and laminarin were administered intraperitoneally once a day for 7 consecutive days before IR.

### 4.2. Surgery of IR

Briefly, as described previously [45], the gerbils used in this study were anesthetized with a mixture of 2–3% isoflurane in 33% oxygen and 67% nitrous oxide using inhalation anesthesia equipment (Harvard Apparatus, Holliston, MA, USA). The bilateral common carotid arteries were exposed through a 2-cm ventral midline incision in their necks and simultaneously occluded for 5 min with non-traumatic aneurysm clips (Yasargil FE 723K, Aesculap, Tuttlingen, Germany). Five minutes later, the clips were removed to restore cerebral blood flow. The restoration of blood flow was directly observed through the retinal arteries, which are branches of the internal carotid arteries, with an ophthalmoscope (HEINE K180, Heine Optotechnik, Herrsching, Germany). The body temperature of each animal was checked and maintained at a normothermic condition (37 ± 0.5 °C) throughout the whole process of the experiment using a heating pad (homeothermic monitoring system, Harvard Apparatus, Holliston, MA, USA). The gerbils subjected to the sham operation received the same IR surgery without occlusion of both common carotid arteries.

### 4.3. Preparation of Brain Sections

As previously described [46], the animals (*n* = 5 in the control group; *n* = 5 at 1 and 5 days post-IR in each sham group; *n* = 7 at 1 and 5 days post-IT in each IR group) were intraperitoneally anesthetized with urethane (1.5 g/kg, Sigma–Aldrich, St. Louis, MO, USA) and perfused with a solution of 4% paraformaldehyde (in 0.1 M phosphate buffer, pH 7.4). Their brains were removed and more fixed (for 6 h) with the same fixative. The fixed brains were cryoprotected by infiltration with a solution of 30% sucrose [in 0.1 M phosphate-buffered saline (PBS), pH 7.4] and coronally sectioned (30 μm thickness) in cryostat (Leica, Wetzlar, Germany).

### 4.4. NeuN Immunofluorescence and FJB Histofluorescence Staining

To evaluate the protection of neurons by pretreated laminarin, NeuN (a marker for neuron) immunofluorescence staining and FJB (a fluorescent marker for neuronal degeneration) histofluorescence staining were performed on the hippocampus at 5 days post-IR. Briefly, as previously described [45], the brain sections were incubated with a solution of mouse anti-NeuN (1:1000, Chemicon, Temecula, CA, USA) overnight at 4 °C. These sections were washed in PBS and reacted with a solution of Cy3-conjugated donkey anti-mouse immunoglobulin G (1:500, Vector Laboratories Inc., Burlingame, CA, USA) for 2 h at room temperature. Regarding FJB histofluorescence staining, the sections were immersed in a solution of 1% sodium hydroxide (Sigma–Aldrich, St. Louis, MO, USA), transferred to a solution of 0.06% potassium permanganate (Sigma–Aldrich, St. Louis, MO, USA) and incubated in a solution of 0.0004% FJB (Histochem, Jefferson, AR, USA). Finally, these stained sections were washed and placed on a slide warmer controlled at about 50 °C for 4 h to be reacted.

Neuroprotection was analyzed as previously described [47]. Five sections containing the hippocampus were selected with a 140-μm interval in each gerbil brain. These sections were anteroposterior from −1.4 to −2.2 mm of the gerbil brain atlas [48]. NeuN^+^ and FJB ^+^ cells were counted as follows. Briefly, digital images of all of the cells were captured in the hippocampus with a fluorescence microscope (BX53) (Olympus, Tokyo, Japan) equipped with a digital camera (DP7) (Olympus, Tokyo, Japan) connected to a PC monitor. Both cells were captured in 200 × 200 μm^2^ at the center of CA1. Each mean cell number was obtained by averaging the total numbers using an image analyzing system (Optimas 6.5) (CyberMetrics, Scottsdale, AZ, USA).

### 4.5. DHE Staining for Superoxide

DHE (Sigma–Aldrich, St. Louis, MO, USA) is the most popular fluorogenic probe to detect intracellular superoxide anions and is used to analyze oxidative stress. We applied DHE staining in the brain sections, as described previously [47]. Briefly, the sections were equilibrated in a Krebs–HEPES buffer (composed of 130 mM NaCl, 5.6 mM KCl, 2 mM CaCl_2_, 0.24 mM MgCl_2_, 8.3 mM HEPES, 11 mM glucose, pH 7.4, etc.) for 40 min at 37 °C. Fresh buffer containing DHE (10 μmol/L) was applied on the sections for 2 h at 37 °C, and DHE was oxidized upon reaction with superoxide to ethidium bromide, which binds DNA in nuclei.

Oxidative stress was analyzed based on the relative fluorescence intensity of DHE. Briefly, as described previously [27], images were captured from CA1 using a BX53 fluorescence microscope (Olympus, Tokyo, Japan) with an excitation wavelength of 520–540 nm. The DHE fluorescence intensity was analyzed using Image-pro Plus 6.0 software (Media Cybernetics, Rockville, MD, USA). The relative intensity was calibrated as a percentage, with the vehicle-sham group, which was designated as 100%.

### 4.6. Immunohistochemistry (IHC)

IHC was done to examine the effects of pretreated laminarin on IR-induced oxidative stress and neuroinflammation. Briefly, as described previously [45], the brain sections obtained at sham, 1 day and 5 days after IR were incubated with a solution of mouse anti-HNE (1:1000, Alexis Biochemicals, San Diego, CA, USA) for lipid peroxidation, a solution of sheep anti-SOD1 (1:1000, Calbiochem, La Jolla, CA, USA) and a solution of sheep anti-SOD2 (1:1000, Calbiochem, La Jolla, CA, USA) for antioxidants, a solution of rabbit anti-IL-1β (1:200, Santa Cruz Biotechnology, Santa Cruz, CA, USA) and a solution of rabbit anti-TNFα (1:1000, Abcam, Cambridge, MA, USA) for the pro-inflammatory response, and a solution of rabbit anti-IL-4 (1:200, Santa Cruz Biotechnology, Santa Cruz, CA, USA) and a solution of rabbit anti-IL-13 (1:200, Santa Cruz Biotechnology, Santa Cruz, CA, USA) for the anti-inflammatory response. These incubated sections were reacted with a solution of biotinylated goat anti-mouse, sheep, or rabbit IgG (1:200, Vector Laboratories Inc., Burlingame, CA, USA) as a secondary antibody and exposed to a solution of avidin–biotin complex (1:300, Vector Laboratories Inc., Burlingame, CA, USA). Finally, these immunoreacted sections were visualized by reacting with a solution of 3,3’-diaminobenzidine tetrahydrochloride (Sigma–Aldrich, St. Louis, MO, USA).

The immunoreactive structure of each HNE, SOD1, SOD2, IL-1β, TNFα, IL-4 and IL-13 was quantitatively analyzed as a relative immunoreactivity (RI). As previously described [45], in short, an image of each immunoreactive structure was captured with an Axio Imager 2 microscope (Carl Zeiss, Oberkochen, Germany) equipped with a digital camera. Each image was calibrated into an array of 512 × 512 pixels, and the immunoreactivity of each structure was evaluated on the basis of optical density (OD), which was obtained after the transformation of the mean gray level of each immunoreactive structure using a formula: OD = log (256/mean gray level). Finally, RI was calibrated as a percentage by using Adobe Photoshop (version 8.0, San Jose, CA, USA) and NIH Image software (1.59). The ratio of RI was calibrated as a percentage, with the vehicle-sham group designated as 100%.

### 4.7. Statistical Analysis

Data are presented as the means ± standard errors of the mean (SEM). All statistical analyses were performed using GraphPad Prism (version 5.0) (GraphPad Software, La Jolla, CA, USA). A multiple-sample comparison was applied to test the IR-related differences between the groups (two-way analysis of variance [ANOVA] and the Bonferroni’s multiple comparison test as a post hoc test using the criterion of the least significant differences). Statistical significance was considered at *p* < 0.05.
